# Lung Protection Strategies during Cardiopulmonary Bypass Affect the Composition of Bronchoalveolar Fluid and Lung Tissue in Cardiac Surgery Patients

**DOI:** 10.3390/metabo8040054

**Published:** 2018-09-21

**Authors:** Raluca G. Maltesen, Katrine B. Buggeskov, Claus B. Andersen, Ronni Plovsing, Reinhard Wimmer, Hanne B. Ravn, Bodil S. Rasmussen

**Affiliations:** 1 Department of Anesthesia and Intensive Care Medicine, Aalborg University Hospital, 9000 Aalborg, Denmark; bodil.steen.rasmussen@rn.dk; 2 Department of Cardiothoracic Anesthesia, Heart Centre, Rigshospitalet, 2100 Copenhagen, Denmark; katrine.buggeskov@gmail.com (K.B.B.); hanne.berg.ravn@regionh.dk (H.B.R.); 3 Department of Forensic Medicine, University of Copenhagen, 2100 Copenhagen, Denmark; clauskrik@gmail.com; 4 Department of Intensive Care, Rigshospitalet, University of Copenhagen, 2100 Copenhagen, Denmark; rplovsing@hotmail.com; 5 Department of Anesthesiology, Hvidovre Hospital, University of Copenhagen, 2650 Hvidovre, Denmark; 6 Department of Chemistry and Bioscience, Aalborg University, 9220 Aalborg, Denmark; rw@bio.aau.dk; 7 Department of Clinical Medicine, School of Medicine and Health, Aalborg University, 9220 Aalborg, Denmark

**Keywords:** ischemia-reperfusion injury, CPB, lung protection, HTK, oxygenated blood, lung biopsy, bronchoalveolar lavage, inflammation, metabolites, NMR

## Abstract

Pulmonary dysfunction is among the most frequent complications to cardiac surgeries. Exposure of blood to the cardiopulmonary bypass (CPB) circuit with subsequent lung ischemia-reperfusion leads to the production of inflammatory mediators and increases in microvascular permeability. The study aimed to elucidate histological, cellular, and metabolite changes following two lung protective regimens during CPB with Histidine-Tryptophan-Ketoglutarate (HTK) enriched or warm oxygenated blood pulmonary perfusion compared to standard regimen with no pulmonary perfusion. A total of 90 patients undergoing CPB were randomized to receiving HTK, oxygenated blood or standard regimen. Of these, bronchoalveolar lavage fluid (BALF) and lung tissue biopsies were obtained before and after CPB from 47 and 25 patients, respectively. Histopathological scores, BALF cell counts and metabolite screening were assessed. Multivariate and univariate analyses were performed. Profound histological, cellular, and metabolic changes were identified in all patients after CPB. Histological and cellular changes were similar in the three groups; however, some metabolite profiles were different in the HTK patients. While all patients presented an increase in inflammatory cells, metabolic acidosis, protease activity and oxidative stress, HTK patients seemed to be protected against severe acidosis, excessive fatty acid oxidation, and inflammation during ischemia-reperfusion. Additional studies are needed to confirm these findings.

## 1. Introduction

Postoperative pulmonary dysfunction is the most common complication to cardiac surgeries with the use of cardiopulmonary bypass (CPB), leading to prolonged hospitalization, increased morbidity, and mortality [1]. The reasons are multifactorial and primarily related to impairments in gas exchange [2]. The main triggers are the CPB circuit with the patients’ blood being exposed to a wide range of synthetic materials which induces a systemic inflammatory reaction and the ischemia-reperfusion injury following weaning from CPB [3,4]. This can lead to postoperative bleeding, infection, and multiple organ dysfunction syndromes (MODs), including acute lung injury and its more severe form, acute respiratory distress syndrome (ARDS) [5,6,7,8]. Alveolar and endothelial damage resulting in formation of pulmonary edema, accumulation of alveolar protein [9,10], and facilitation of inflammatory cell sequestration have previously been confirmed in lung tissue biopsies and bronchoalveolar lavage fluid (BALF) [11]. The consequence is a thickened alveolar-endothelial barrier with compromised gas exchange between alveoli and the blood [12].

We have previous demonstrated that CPB significantly impact the levels of blood metabolites with fatty acids, phospholipids, ketones, and several amino acids were predictive of the degree of lung tissue injury commonly observed on the second to third postoperative day [13,14]. Although these studies provided insight into the systemic changes occurring during progression to lung injury after CPB, there is still a need for knowledge regarding the pulmonary molecular changes during surgery.

Increasing evidence indicates that lung tissue protection by pulmonary artery perfusion with blood [15,16,17,18,19,20,21] or a preservation solution [22,23,24,25] such as hypothermic histidine-tryptophan-ketoglutarate (HTK [24,25,26]) during CPB may attenuate postoperative hypoxemia by supplying metabolic substrates to the lung tissue exposed to re-perfusion [27]. Hypothermic HTK is used as a multi-organ preservation solution for organ transplantation, including the lungs [25,28,29,30], in addition to its use as a cardioplegia solution. Its buffer system is thought to withdraw extracellular sodium and calcium, together with buffering of the extracellular space by means of histidine and histidine-hydrochloride, thus prolonging the period during which organs tolerates interruption in blood supply [31]. It has been found that HTK solution attenuated pulmonary edema until 12 h post-perfusion when compared to both saline and the low-potassium dextran preservation solution in an experimental rat model of ex vivo lung perfusion [32]. In the randomized Pulmonary Protection Trial of adult patients undergoing elective cardiac surgery [26], we found that postoperative arterial oxygenation on the first postoperative day was slightly better following pulmonary artery perfusion with normothermic oxygenated blood during CPB compared to pulmonary artery perfusion with hypothermic HTK solution and standard regimen with no perfusion. However, the baseline arterial oxygenation was higher in the patients receiving oxygenated compared to the two other groups. The main aim of this study is to elucidate the pathophysiological changes within lungs in more details in the three groups of patients included in the Pulmonary Protection Trial.

BALF and lung biopsies were collected from patients included in The Pulmonary Protection Trial [26] before and after CPB. Both histological, alveolar cell, differential count of leucocytes, and metabolites were investigated. Profound histological, cellular, and metabolic changes were identified in the lung tissue and BALF after CPB in all patients, regardless of their treatment allocation. While all patients presented increased inflammatory cells, metabolic acidosis, protease activity, and oxidative stress in BALF, HTK patients seem to be more protected against severe acidosis, excessive fatty acid oxidation, and inflammation following an ischemia-reperfusion period.

## 2. Materials and Methods 

### 2.1. Study Population

The Pulmonary Protection Trial was conducted in accordance with the Declaration of Helsinki, and approved by the Committees on Biomedical Research Ethics of The Capital Region of Denmark (approved number: H-1-2012-024), the Danish Medicines Agency (approved number: 2012024017, EudraCT number: 2011-006290-25, protocol code: 4141), and the Danish Data Protection Agency (approval number: 2011-41-7051). The study was registered at ClinicalTrials.gov (NCT01614951) and monitored by Copenhagen University Hospital Good Clinical Practice Unit. The rationale for and design of the trial and the primary results have been published [26,33].

In the original study, The Pulmonary Protection Trial [26], 90 patients scheduled for elective coronary artery bypass grafting, aortic valve replacement or a combination of both procedures at Rigshospitalet, Copenhagen University Hospital, Denmark were included. The BALF and lung biopsy procedures was first added to the trial halfway due to the complexity of the trial and the number of involved trialist did not allow us to add the extra procedures until that point. The current study is therefore a sub-study of the original Pulmonary Protection Trial [26] including patients from whom BALF (47 patients) and lung biopsy (25 patients) specimens were collected. All subjects gave their informed consent for inclusion before they participated in the study. Inclusion criteria were adults (≥18 years) with chronic obstructive pulmonary disease (COPD). Exclusion criteria were individuals with previous heart or lung surgeries, previous thoracic radiation, left ventricular ejection fraction <20%, tracheal intubation or medically treated pneumonia prior to surgery. Patients were randomized to three groups with 30 patients in each; i.e., pulmonary artery perfusion with oxygenated blood (‘oxygenated’), pulmonary artery perfusion with HTK (‘HTK’), and standard regimen with no perfusion of the pulmonary artery (‘standard CPB’), respectively.

### 2.2. Bronchoalveolar Lavage Fluid

Bronchoalveolar lavage fluid (BALF) was performed in a sub-segment of the right middle lobe following induction of anesthesia before surgery and again at skin closure from 47 out of the 90 patients (standard CPB, *n* = 19; oxygenated, *n* = 15; HTK group, *n* = 13) using standardized procedure [34]. Lidocaine gel (20 mg/g) was applied to the tip of the bronchoscope before insertion and samples were collected in a standardized fashion [34]. In brief, three successive 50 mL aliquots of isotonic saline (at 37 °C) were instilled, each aspirated immediately with low negative suction pressure (<100 cm H_2_O), and pooled into a sterile glass container on ice to obtain one BALF specimen. Pooled non-filtered and non-centrifuged BALF was immediately used to enumerate the total number of nucleated cells per mL of retrieved BALF on an automated hematology analyzer (Sysmex XE-2100, Sysmex Corp., Kobe, Japan) and differential cell counts were performed manually after cytocentrifugation (Cytospin 4 Cytocentrifuge, Thermo Fisher Scientific, Hvidovre, Denmark) with subsequent May-Grunwald-Giemsa staining (Sysmex SP-1000i, Sysmex Europe GmbH, Hamburg, Germany). Differential cell counting was performed for ~200 cells according to standard morphological criteria by two independent hematology technicians and data is presented as cells per mL BALF. Cytospin samples containing >20% smudge cells and/or artefacts were excluded from analysis; five samples were excluded according to these criteria.

For metabolite analysis, pooled non-filtered BALF was centrifuged in tubes containing a carrier protein (1% BSA in PBS) at 3500 rpm and 4 °C for 15 min and the supernatants were kept at −80 °C until analyzed on a BRUKER AVIII-600 MHz nuclear magnetic resonance (NMR) spectroscopy (BrukerBioSpin, Rheinstetten, Germany) equipped with a cryogenically cooled, triple-resonance CPP-TCI probe. 

BALF samples were thawed for 30 min at 4 °C and centrifuged for 5 min (4 °C, 12,100× *g*) to remove cells. Aliquots of 500 μL of supernatant were mixed with 100 μL D_2_O phosphate buffer (0.2 M Na_2_HPO_4_/NaH_2_PO_4_, pH = 7.4, in 99% ^2^H_2_O) to minimize variation in pH and to provide a field-frequency lock. Throughout the whole procedure samples were kept on ice. The prepared samples were transferred to a 5 mm NMR tube and spectra were recorded at a temperature of 298.1 K. ^1^H-NMR spectra were acquired using a T_2_-filtered one-dimensional Carr-Purcell-Meiboom-Gill (CPMG) [35] pulse sequence with water suppression, as previously described [13,14]. Spectra acquisition was controlled using the TopSpin 3.1 software (Bruker BioSpin, Germany). 

### 2.3. Lung Biopsy

Two lung biopsies were harvested from 25 out of the 90 patients (standard CPB, *n* = 9; oxygenated, *n* = 9; HTK group, *n* = 7) from either the middle lobe or lingual at initiation of CPB and again after weaning from CPB. The tissue was fixed in 10% formaldehyde solution followed by embedding in paraffin, and 4 μm sections were stained with hematoxylin and eosin for light microscopy. A histopathological score of acute lung injury was evaluated by a pathologist in accordance with the Mikawa’s method [36]. The score graded each item on a 5-point scale (0, minimal damage; 1, mild damage; 2, moderate damage; 3, severe damage; 4, maximal damage) based on (a) congestion in the alveolar capillaries, hemorrhage, (b) neutrophilic infiltration or aggregation in airspace or vessel wall, (c) thickness of the alveolar septae, and (d) hyaline membrane formation [37,38].

### 2.4. Data Analysis

CPMG spectra were manually phased and baseline corrected, calibrated to the alanine doublet at 1.48 ppm in TopSpin 3.1 and reduced to integrals of 0.001 ppm bin widths excluding the water region (4.5–5.1 ppm) in the AMIX (Analysis of MIXtures v. 3.9.10, Bruker BioSpin, Karlsruhe, Germany) software. The remaining spectral data was exported to MATLAB (R2016a, MathWork) and was subsequently normalized to total sum of the spectral intensity, log transformed, and mean centered. Multivariate unsupervised pattern recognition analysis was performed using the PLS Toolbox 6.5 (Eigenvector Research, Wenatchee, WA, USA). Unsupervised principal component analysis (PCA) was carried out to identify possible outliers and trends in the data. Data was visualized by plotting the scores of the first two principal components (PC1 and PC2). Loadings plot was used to identify the significant spectral regions responsible for sample clustering in the scores plot.

Metabolites were assigned based on our previous study [14] and the Human Metabolite Database [39] while metabolite quantification was determined using peak fitting option in the MNOVA Mestrelab Research v.12 software. The statistical analysis was performed in the IBM^®^ SPSS Statistics software (v. 24, SPSS Inc., Armonk, NY, USA).

To be able to determine differences between lung histopathological score, differential cell counts, and metabolites measured before and after surgery, we first employed a Shapiro-Wilk test to verify if the data follows a normal distribution. Accordingly, pair *t*-test or the nonparametric test Mann-Whitney-Wilcoxon was applied. Also, to detect differences between paired BALF samples collected within the three groups before and after the surgery a factorial two-way analysis of variance (ANOVA) with Tukey’s post-hoc or the Kruskal Wallis test was used for multiple comparisons. Overall fold changes were calculated by comparing after versus before CPB values. Additionally, the effect of treatment was assessed by calculating FC differences between oxygenated versus standard CPB and HTK versus standard CPB groups from sample collected after CPB only. A calculated two-tailed *p*-value of less than 0.05 was considered statistically significant. Bar plots with mean ± standard deviation (SD) representing 95% confidence level was used to visualize the data.

## 3. Results

Of the 90 patients included in The Pulmonary Protection Trial, BALF was collected before and after CPB in 47 patients and lung biopsies in 25 patients. Distribution to the lung protection and standard regimens, patient characteristics, duration of surgery, and arterial oxygenation are provided in Table 1. There was no statistic significant difference between the three groups of patients.

### 3.1. Lung Histological and Cellular Changes during Surgery

**Figure 1:**
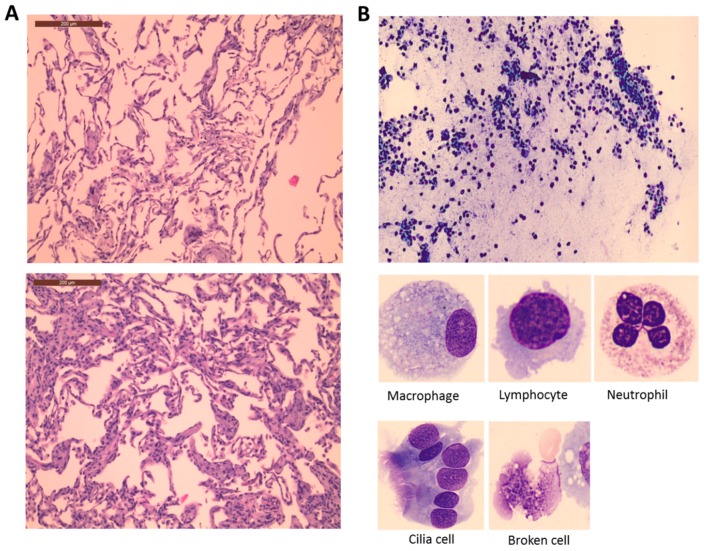
Lung histological (**A**) and BALF cellular (**B**) changes during surgery. (**A**) Image from a patient before (above) and after CPB (below) presenting all possible outcomes on the 5-point lung injury scale (congestion in the alveolar capillaries, hemorrhage, neutrophilic infiltration or aggregation in airspace or vessel wall, thickness of the alveolar septae, and hyaline membrane formation). Additional information about the lung injury score is provided in Section 4.3. (**B**) Representative image of a BALF sample taken from a patient after CPB. The morphology of the most common cells identified is shown below.

Two lung tissue samples were obtained from 25 patients before and after CPB. Histopathological tissue changes (Figure 1A) and slightly increases in the total acute lung injury scores (Table 2) together with thickened alveolar sepae, alveolar congestion, and neutrophil infiltration were observed postoperatively, irrespective of treatment allocation.

Two BALF samples were obtained from 47 patients before and after CPB. Figure 1B shows representative image of the histological examination of BALF in one of the patients. Several different nucleated cells (e.g., white blood cells including neutrophils, lymphocytes, and macrophages; other cells including cilia) and non-nucleated cells (e.g., broken nucleated cells, smudge, cellular fragments, etc.) were present in post-CPB samples, indicating increased permeability of the alveolar membrane with cell influx from the blood stream initiated by an inflammatory response during surgery (Table 3). No statistically significant difference was observed in the cellular levels across the three groups. The picture that emerges from post-CPB BALF cellular count is one of inflammation with increased total number of nucleated white blood cells, cilia and non-nucleated cells, and a decreased number of macrophages and lymphocytes. A slightly increased number of neutrophils were observed postoperatively; however, the change was statistically insignificant.

### 3.2. Lung Metabolite Profiles

**Figure 2:**
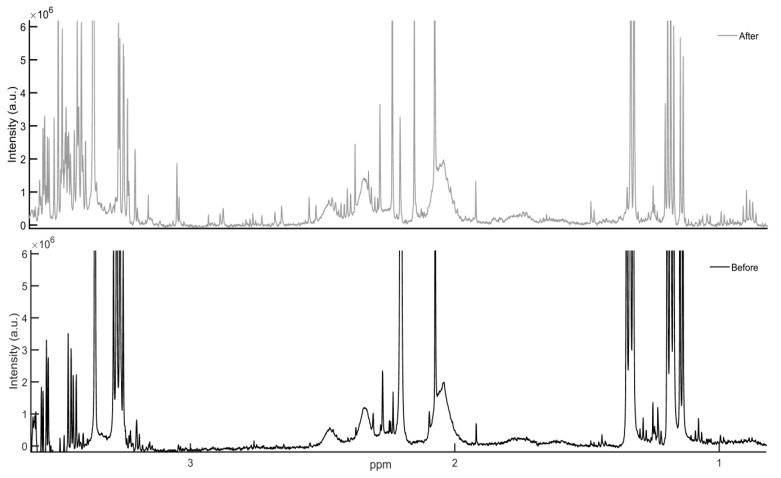
600 MHz ^1^H-NMR CPMG spectra of metabolic intensities in one patient’s bronchoalveolar lavage fluid obtained before (black) and after CPB (grey). The position of a signal is a characteristic of a given compound, while the area under the signal is proportional to the compound’s concentration. An increased number is observed in the signals of sample collected after CPB.

Figure 2 illustrates a region of one-dimensional ^1^H-NMR spectra of BALF samples from one patient, collected before (black) and after (grey) CPB. Visual inspection of both spectra revealed a larger number of intensities in the post-CPB sample, indicating an increased number of metabolites. 

**Figure 3:**
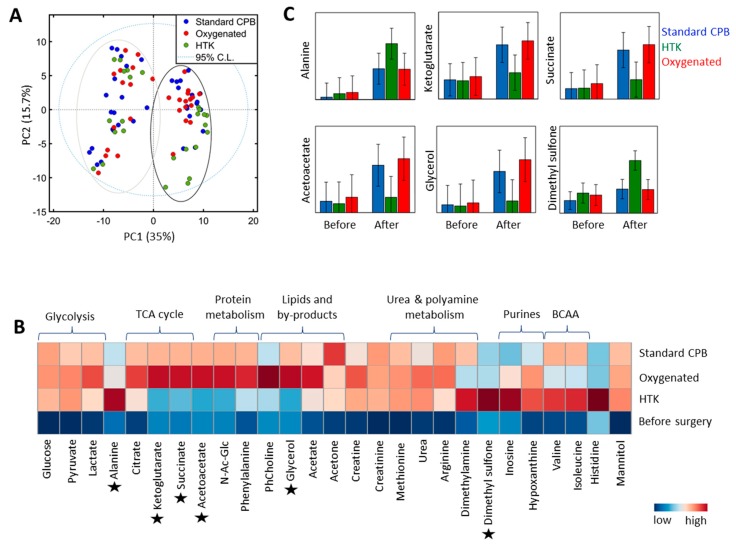
Lung metabolic profiles. (**A**) Principal component analysis (PCA) before (−PC1 axis) and after CPB (+PC1 axis) colored by the treatment received (standard CPB, blue; oxygenated blood, red; HTK, green). (**B**) Averaged heat-map representation of samples collected before (bottom raw) and after CPB (second to forth raw). Data are normalized to total intensity and auto-scaled, colored coded according to the levels of metabolites identified at a particularly time point and group; e.g., blue represent lower concentrations of metabolites, while red represents metabolites in which concentration increased post-CPB. Six metabolites presenting different trends across the groups are marked (*) and further displayed in (**C**) with mean and 95% confidence intervals. TCA: tricarboxylic cycle acid; BCCA: branch chain amino acid.

Unsupervised principal component analysis (PCA) revealed that the main variation in patients’ metabolite profiles is due to the ischemia-reperfusion itself (35%), as demonstrated by sample clustering along the first principal component (PC1) (Figure 3A). The second main variation (15.7%) is due to the treatment received during CPB (PC2); with HTK patients clustering separately compared to the overlapping standard CPB and oxygenated blood groups. In order to avoid loss of valuable information, metabolite changes related to both CPB and treatment regimens received will be presented (Table 4). In general, similar metabolite trends were observed post-CPB in all patients irrespective of the intervention. For example, 3 fold to 10 fold change (FC) increases were observed in the concentration of metabolites involved in glycolysis (glucose, lactate, and pyruvate), while metabolites involved in the urea cycle and nitric oxide (NO) production (urea, arginine, and methionine) were markedly elevated in all patients with FC ≥ 20 times (*p* < 0.0001), indicating significant changes in these pathways during CPB. For some metabolites, patients receiving either the standard regimen or pulmonary perfusion with oxygenated blood appeared to have similar trends (Table 4 and Figure 3B). For example, the NO inhibitor dimethylamine (DMA) was found to be elevated in all patients after CPB; however, the standard regimen and pulmonary perfusion with oxygenated blood group presented less increases compared to the HTK-receiving group (FC = 2.1, *p* = 0.004). Augmented levels of metabolites involved in protein catabolism (alanine, valine, isoleucine, creatine, creatinine, and phenylalanine) were also detected in all patients (FC ≥ 2, *p* ≤ 0.01), with highest alanine values in the HTK-receiving group. Dimethyl sulfone, known to have anti-inflammatory activity, and the by-products of purine metabolism hypoxanthine and inosine, known to be linked to oxidative stress formation, were also elevated in all patients post-CPB. However, while no significant difference was observed in the levels of dimethyl sulfone in the standard versus oxygenated blood groups post-CPB (FC = 0.9, *p* = 0.8, Figure 3C), patients receiving HTK presented a 2.1 FC increase (*p* = 0.004) compared to the standard regimen. Finally, some metabolites showed changes only in the standard and oxygenated group post-CPB, while their levels remained unchanged in the HTK-receiving group or example, while glycerol concentration increased in both the standard regimen and oxygenated blood groups following CPB (FC= 1.3, *p* = 0.7), glycerol values remained unchanged in the HTK group (*p* = 0.20). Similar trends were observed with respect to acetoacetic acid (*p* = 0.37) and the TCA cycle metabolites 2-ketoglutarate (*p* = 0.51) and succinate (*p* = 0.20) in the HTK-receiving patients, indicating a preservation of the molecular pathways involved in the synthesis of these metabolites.

## 4. Discussion

Lung injury following cardiac surgeries with CPB is a common and potentially serious event that may lead to ARDS [5,6,7,8]. Although various factors have been found to influence the onset of lung injury and the progression into hypoxemic respiratory failure, the associated molecular alterations remain poorly characterized. In this study, we have characterized lung morphological, cellular, and metabolic changes following ischemia-reperfusion. These findings may add new insights into the molecular mechanism of lung injury during CPB and may play a key role in understanding the progression towards respiratory failure, thus enabling development of potential treatment modalities. 

### 4.1. Lung Histological and Cellular Changes during Surgery

It has previously been shown in animal models that CPB is directly linked to vascular permeability and alveolar flooding [27,40]. In 1967 Tilney and co-workers [41] performed histological examination of lung tissue from deceased patients following prolonged CPB and found hypercellular and thickened alveolar septae, fresh hemorrhage, and edema within alveoli with prominent hyaline membranes. In 1972 these changes were also found in patients surviving cardiac surgery [42]. Our results confirm the histopathological evidence of acute lung injury immediately after CPB, with alveolar congestion, neutrophilic infiltration, and thickened alveolar septae in all patients regardless of the treatment received.

The observed increase in alveolar cellularity confirmed the trans-endothelial migration of inflammatory cells following surgery. Only a few human studies have described cellular changes in BALF following CPB. Frass and colleagues [43] found no significant changes in BALF differential cell count while Marsh’s group [44] found a peak in cellularity on day two. This was mainly driven by an increase in neutrophils associated with a corresponding fall in macrophages. Zhang and co-workers found increased neutrophil concentration in BALF peaking at 12 h post-CPB [45], thus explaining why the neutrophil count was unchanged two hours after CPB in our study.

### 4.2. Lung Metabolite Profiles

Overall, the metabonomics analysis revealed changes in the levels of metabolites involved in energy metabolism, protein catabolism, lipids, purines, and polyamine metabolism. Compared with morphological and cellular events, some metabolites showed significant differences between patients receiving pulmonary perfusion with HTK compared to no pulmonary perfusion during CPB.

We found that metabolites involved in glycolysis were elevated in all patients after CPB. Interestingly, lactate was similarly increased in all patients and this was especially noteworthy in the patients receiving oxygenation blood perfusion, where a less hypoxic environment in the lungs was expected. In addition, elevated levels of acetate were identified in all patients. Abnormal BALF lactate [46] and acetate [47] levels have previously been linked to the activation of inflammatory cells during lung injury; hence, our findings indicate increased anaerobic metabolite concentrations (lactate and acetate) and inflammation (BALF cellularity) in all patients during ischemia-reperfusion injury. An interesting finding is that the levels of acetoacetic acid were only elevated in the oxygenated and standard CPB patients after surgery. Acetoacetic acid is known to be produced from acetyl-CoA by breakdown of fatty acids and complex lipids. Our data shows that the levels of the components of cellular membranes, phosphocholine and glycerol were augmented after CPB in the oxygenated group and in the standard group, but not in the HTK group. Hence, the results indicate that HTK patients were protected from excessive fatty acid oxidation in comparison to the other groups. Our results are supported by the systematic review performed by Edelman and co-workers [48], where tryptophan in the HTK solution was described as stabilizing cell membranes while histidine buffers the ongoing metabolic acidosis during ischemia.

Several amino acids were found to be highly elevated at the end of surgery in all patients. Free amino acids in BALF samples have been previously linked to increased protease activity in the alveoli [49,50] and lung injury [47], hence our results might indicate the presence of ischemia-reperfusion injury during CPB in all patients.

The by-products of purine metabolism, hypoxanthine and inosine, were also found to be elevated at the end of surgery. These metabolites are known to accumulate during ischemia due to reduced oxygen and energy supply. Hypoxanthine has been previously found to be elevated in ARDS patients and its presence in the alveoli has been linked to the generation of alveolar oxygen superoxide and hence, of oxidative stress [51,52]. Our findings suggest increased oxidative stress during surgery, with no differences between the three groups. It is well known that reduced oxygen concentrations in the lung enhance the activity of polyamine metabolism [53]. When compared with preoperative concentrations, the polyamine metabolites methionine and arginine were found in excess. Normal epithelial cells synthesize polyamines and the lungs are in fact endowed with a much higher polyamine uptake system than other organs, in order to protect the tissue against oxidative stress [53]; however, such excesses (FC > 20) may suggest toxic reactions of the cells and their surroundings. Excess polyamine metabolites were previously found to induce disruption of the barrier function of the epithelium, and lead to pulmonary edema with subsequent invasion of inflammatory cells [53]. Hence, our data might reflect increased oxidative stress and a possible cellular disruption, facilitating molecular leakage into the alveolar space, regardless of the pulmonary artery perfusion strategy used. Apart from being involved in polyamine synthesis, arginine is at a crossroad for multiple other pathways and it is a common substrate for arginase and nitric oxygen species via urea cycle. Urea was also found elevated (19.9-fold), indicating an increased arginase activity. Dimethylamine, a potent inhibitor of NO synthesis [54] was found to be increased 3.3-fold. Its increases might indicate accumulation of reactive oxygen and nitrogen species, contributing to lung tissue damage in all patients. 

In line with higher oxidative stress, the keto acid of the TCA cycle metabolite 2-ketoglutaric acid and its converted metabolic form, succinate, were found to be elevated in the oxygenated and standard CPB patients, but not in the HTK group after ischemia-reperfusion. 2-Ketoglutaric acid has a role in detoxification of reactive oxygen species (ROS) [55] and it is known to improve ATP production during reperfusion [41]. Increased levels of these metabolites in standard and oxygenated patients might indicate that they are being synthesized to balance the oxidant-antioxidant defense in these patients. Since HTK contains 2-ketoglutaric acid [27], a plausible explanation for its insignificant increase among HTK patients might be that the cardioplegia provided the necessary amount of protection. Finally, dimethyl sulfone, a metabolite with anti-inflammatory activity, was found to be elevated after ischemia-reperfusion in all patients. Because the HTK patients presented a 2.1-fold increase in its levels compared to the standard CPB group, it might possibly indicate that HTK provides some protection against the activity of inflammatory cells. These findings are in line with the study by Warnecke et al. [29] where piglets’ lungs were perfused with three different preservation solutions followed by BALF, with the lowest post-perfusion cell count in the piglets receiving HTK solution.

### 4.3. Strengths and Limitations

Although this study is the first of its kind, there are some limitations to acknowledge. Firstly, in this sub-study of the original Pulmonary Protection Trial [26], only a limited number of the 90 patients have contributed to the present results; 47 patients with BALF and 25 of these patients with lung biopsies. Selection of patients was based on organizational logistics and not patient characteristics. Another limitation is the use of a semi-quantitative grading system to obtain the histopathological scores of tissue damage. Quantitative stereological analysis by the use of more robust unbiased morphometry might improve the results, but this would require a different and more elaborate study design regarding tissue sampling and evaluation, which is difficult to apply in a clinical study. In addition, since the BALF samples have been diluted with isotonic saline before their collection, important information from low concentration metabolites may be missing. Also, lidocaine has been used during the BALF procedure. Spectroscopically, lidocaine peaks spanned several metabolite-rich spectral regions, and hence, the number of detected metabolites was reduced, resulting in possible loss of valuable information. In addition, mannitol peaks were also overlapping metabolites, especially in the 4–3 ppm region, hence several metabolites were unable to be detected and quantified.

## 5. Conclusions

In this sub-study of The Pulmonary Protection Trial we found molecular and histological changes in the lung tissue and BALF following CPB with slight alveolar congestion, thickened alveolar septae, neutrophilic infiltration, increased BALF inflammatory cells, metabolic acidosis, increased protease activity, and oxidative stress. Pulmonary perfusion with HTK during CPB seemed to protect against severe acidosis, excessive fatty acid oxidation, and inflammation during lung ischemia-reperfusion injury. Additional studies are needed to confirm the results and to further elucidate the deranged mechanisms involved in the progression to ARDS.

## Figures and Tables

**Table 1:** Patient characteristics.

	HTK/Oxygenated/Standard CPB
Patient no.	BALF: 13/15/19 (Lung biopsy: 7/9/9)
Age-years (mean ± SD)	72 ± 9; 71 ± 12; 70 ± 8
Gender (male/female)	10/3; 12/3; 17/2
Cardiopulmonary bypass time, min. (mean ± SD)	96 ± 22; 104 ± 63; 107 ± 52
Aortic cross-clamp time, min. (mean ± SD)	61 ± 13; 57 ± 44; 67 ± 42
End-CPB PaO_2_/FiO_2_ (kPa) (mean ± SD)	29 ± 10; 32 ± 10; 27 ± 11

Abbreviation: BALF, bronchoalveolar lavage fluid; HTK, histidine-tryptophan-ketoglutarate; CPB, cardiopulmonary bypass; SD, standard deviation; min., minutes; PaO_2_/FiO_2,_ partial pressure of oxygen/fraction of inspired oxygen ratio in kPa.

**Table 2:** Lung histopathological changes from before and after CPB.

Lung Tissue	Groups	Before			After			Before vs. After	Time-Group
		Mean	Min	Max	Mean	Min	Max	*p*-Value	*p*-Value
Alveolar congestion	Control	1.1	1	2	1.2	1	2	0.06 *	0.62
	HTK	1.0	1	1	1.3	1	2		
	O2	1.1	1	2	1.3	1	2		
Heamorhage	Control	0.4	0	2	0.4	0	2	0.72	0.64
	HTK	0.1	0	1	0.3	0	1		
	O2	0.2	0	1	0.2	0	1		
Neutrophilic infiltration	Control	1.1	1	2	1.6	1	2	<0.001	0.32
	HTK	1.1	1	2	1.5	1	2		
	O2	1.2	1	2	1.3	1	2		
Thickness of alveolar wall hyaline membranes	Control	1.4	1	3	1.6	1	3	0.01 *	0.58
	HTK	1.6	1	3	2.1	1	3		
	O2	1.5	1	3	1.6	1	3		
Total score	Control	4.1	3	6	4.6	3	7	0.001	0.36
	HTK	3.7	3	5	5.1	3	7		
	O2	3.9	3	7	4.3	3	7		

Pair *t*-test or its corresponding non-parametric test (*) was used to identified differences between paired samples (Before vs. After CPB) and factorial two-way ANOVA to detect differences between patients as a consequence of surgery and treatment received (Time-Group interaction). A *p*-value < 0.05 was considered significant. Data is presented as means, minimum (min) and maximum (max) values.

**Table 3:** Results of BALF cellular count in samples collected before and after CPB.

BALF Cells	Group	Before			After			Before vs. After	Time-Group
		Mean	Min	Max	Mean	Min	Max	*p*-Value	*p*-Value
Total white blood cells	Control	157,435	22,000	990,000	239,777	52,000	1,020,000	0.015 *	0.59
	HTK	163,846	9000	401,000	173,615	48,000	415,000		
	O2	205,071	43,000	504,000	328,333	74,000	1,185,000		
Neutrophils	Control	18.5	0	89	23.9	1	145	0.44	0.79
	HTK	20.1	2	79	19.5	2	110		
	O2	21.0	1	164	27.0	0	81		
Lymphocytes	Control	7.9	1	25	4.1	0	18	0.007 *	0.78
	HTK	17.5	0	86	13.8	0	46		
	O2	10.9	0	54	10.1	0	50		
Eosinocytes	Control	0.9	0	3	0.8	0	5	0.84	0.96
	HTK	0.9	0	3	0.9	0	6		
	O2	2.4	0	21	2.2	0	17		
Macrophages	Control	161.2	3	274	119.7	12	225	0.02 *	0.26
	HTK	147.9	42	265	158.2	37	227		
	O2	170.4	12	216	135.5	10	217		
Other cells including cilia	Control	12.4	0	89	54.1	0	272	0003 *	0.19
	HTK	22.0	0	230	24.3	0	122		
	O2	5.1	0	41	30.9	0	176		
Non-nucleated cells including broken cells and smudge	Control	58.4	3	471	98.1	2	293	0.05 *	0.63
	HTK	51.8	3	184	48.2	6	221		
	O2	36.9	1	278	65.7	2	200		

Pair *t*-test or its corresponding non-parametric test (*) was used to identified differences between paired samples (Before vs. After CPB) and factorial two-way ANOVA to detect differences between patients as a consequence of surgery and treatment received (Time-Group interaction). A *p*-value < 0.05 was considered significant.

**Table 4:** Metabolite profiles before and after CPB.

BALF Metabolites	Before CPB						After CPB						CPB Effect		Treatment Effect				
	Control		HTK		O2		Control		HTK		O2		Time (After vs. Before)		Time * Group	HTK/Control		O2/Control	
	Mean	SD	Mean	SD	Mean	SD	Mean	SD	Mean	SD	Mean	SD	FC	*p*-Value	*p*-Value	FC	*p*-Value	FC	*p*-Value
Glycerol	31.1	34	33.4	43	36.8	40	143.3	37	46.6	41	185.4	38	-	-	0.11	0.3	0.029	1.3	0.7
Phosphocholine	7.7	7	5.1	9	8.0	8	18.6	7	14.5	8	37.0	8	2	<0.0001	0.7	0.9	0.6	2.2	0.3
*N*-Acetyl-Glucosamine	1.7	2	1.8	2	2.9	2	9.6	2	3.3	2	11.2	2	-	-	0.05	0.3	0.013	1.1	0.8
Acetate	9.0	1	9.6	2	7.6	1	13.7	1	11.4	2	13.9	1	1.6	<0.0001	0.9	0.9	0.3	1	0.9
Acetoacetic acid	7.5	6	7.2	8	9.8	7	29.3	7	10.6	7	34.2	7	-	-	0.14	0.3	0.027	1.1	0.8
Acetone	5.6	2	4.6	3	6.2	2	15.8	2	10.5	2	10.7	2	2	<0.0001	0.9	0.6	0.3	0.7	0.3
Glucose	8.8	9	12.1	12	11.1	11	83.4	10	66.3	11	71.9	11	10	<0.0001	1.0	0.9	0.7	0.9	0.7
Lactate	70.6	19	73.8	24	83.2	22	224.0	21	178.0	23	228.1	21	2.9	<0.0001	0.4	0.8	0.2	1	0.9
Pyruvate	0.9	1	1.4	1	1.9	1	7.6	1	7.3	1	7.5	1	6.2	<0.0001	1.0	1	1	1	0.9
Alanine	1.1	2	2.3	3	2.8	3	11.0	3	22.1	3	10.9	3	7.9	<0.0001	0.05	1.8	0.13	1	1
Citrate	1.9	1	3.8	1	4.6	1	9.6	1	7.3	1	9.7	1	2.4	<0.0001	0.7	0.8	0.3	1.1	0.8
2-Ketoglutarate	12.3	5	14.0	6	13.9	6	33.9	6	16.0	6	38.1	6	-	-	0.02	0.5	0.005	1.1	0.8
Succinate	4.0	3	5.1	4	5.5	3	16.9	3	7.5	3	19.5	3	-	-	0.08	0.4	0.025	1.1	0.8
Creatine	0.4	1	0.7	1	0.6	1	3.0	1	3.0	1	3.6	1	5.8	<0.0001	0.7	1	1	1.2	0.7
Creatinine	2.3	2	1.8	2	3.5	2	18.8	2	14.9	2	15.5	2	6.5	<0.0001	0.8	0.8	0.5	0.8	0.5
Isoleucine	1.1	1	1.4	1	0.8	1	3.9	1	3.3	1	2.3	1	2.3	0.0004	0.3	0.9	0.4	0.7	0.2
Valine	1.6	1	2.1	1	1.9	1	5.1	1	5.1	1	3.5	1	2.2	0.0001	0.07	0.9	0.7	0.7	0.2
Urea	8.8	6	4.3	8	7.2	7	39.5	7	44.2	7	47.2	7	19.9	<0.0001	0.6	1.4	0.3	1.2	0.4
Arginine	2.7	11	2.2	14	2.8	13	74.6	12	50.2	14	69.3	13	28.1	<0.0001	0.5	0.7	0.5	1	0.9
Methionine	0.2	3	0.0	3	0.0	3	13.2	3	12.5	3	12.7	3	88.6	<0.0001	1.0	1.1	0.8	1	1
Dimethylamine	0.4	0	0.6	0	0.3	0	1.7	0	2.0	0	1.1	0	3.3	<0.0001	0.14	1.2	0.5	0.6	0.2
Dimethyl sulfone	3.3	1	3.9	1	4.1	1	6.0	1	11.7	1	5.4	1	-	-	0.004	2.1	0.004	0.9	0.8
Histidine	0.1	29	0.0	36	0.5	33	3.0	31	250.8	35	3.2	32	-	-	<0.0001	67.8	0.0002	1.1	0.9
Phenylalanine	22.8	4	18.2	6	23.7	5	36.0	5	26.8	5	34.1	5	2	0.01	0.3	0.7	0.14	0.9	0.5
Inosine	4.5	1	4.2	1	3.4	1	5.1	1	6.2	1	4.9	1	1.3	0.008	0.5	1.2	0.4	0.9	0.5
Hypoxanthine	4.6	1	4.4	2	4.4	2	9.0	1	10.4	2	9.6	1	3.3	0.0002	0.9	1.1	0.7	1	1
Mannitol	0.0	0	0.0	0	0.0	0	687.7	69	675.4	76	624.8	71	90	<0.0001	0.9	1	1	0.9	0.6

Because several metabolites were without any signal in samples collected before CPB, their mean concentrations were below the limit of NMR detection. Mean fold change (FC) were calculated as the ratio between the metabolites measured (1) before and after CPB (CPB effect), and (2) by comparing the standard regimen versus oxygenated blood group and the standard CPB regimen versus HTK group after CPB (Treatment effect). “-” these metabolites have been found different among groups post-CPB, and hence, FC and *p*-values for the before and after analysis are not provided to avoid misinterpretation. To assess the effect of CPB on BALF metabolites the pair *t*-test or its corresponding non-parametric test was applied, while for the effect of treatment received the factorial two-way ANOVA (Time-Group interactions) and its corresponding Tuckey’s post-hoc test for group comparison was used (HTK/Control; O_2_/Control). A two-tailed *p*-value ≤ 0.05 was considered significant.
